# A cross-sectional survey of stigma towards people with a mental illness in the general public. The role of employment, domestic noise disturbance and age

**DOI:** 10.1007/s00127-021-02111-y

**Published:** 2021-07-16

**Authors:** S. C. C. Oudejans, M. E. Spits, J. van Weeghel

**Affiliations:** 1 Mark Bench, Rhôneweg 16, 1043AH Amsterdam, The Netherlands; 2 Department of Psychiatry, Amsterdam Institute for Addiction Research, Amsterdam UMC, Amsterdam, The Netherlands; 3 Dutch Addiction Association, Amersfoort, The Netherlands; 4 Phrenos Center of Expertise, Utrecht, The Netherlands; 5 Tranzo Scientific Center for Care and Wellbeing, School of Social and Behavioural Sciences, Tilburg University, Tilburg, The Netherlands

**Keywords:** Stigma, Social distance, Housing, Employment, Age, Mental illness, Psychotic disorder, Depressive disorder, Mental health knowledge, Contact

## Abstract

**Introduction:**

Stigmatization impedes the social integration of persons recovering from mental illnesses. Little is known about characteristics of the stigmatized person that lessen or aggravate public stigma.

**Purpose:**

This study investigates which characteristics of persons with mental illnesses (i.e. with a depression or a psychotic disorder) might increase or decrease the likelihood of public stigma.

**Methods:**

Over 2,000 adults read one of sixteen vignettes describing a person with a depressive disorder or a psychotic disorder and answered a set of items measuring social distance.

**Results:**

The person who was employed (vs. unemployed), or whose neighbors did not experience domestic noise disturbance (vs. disturbance) elicited significantly less social distance. Also persons with a depressive disorder elicited less social distance, vs. persons with a psychotic disorder.

**Conclusion:**

Employment and good housing circumstances may destigmatize persons coping with mental illnesses. Mental health and social services should encourage paid employment, quality housing and other paths to community integration.

## Introduction

Stigma is a major concern for people living with a mental illness. The term stigma is applied when the following elements co-occur: (a) a distinction of labelling of human differences is made (such as skin colour, but also receiving mental health treatment), (b) dominant cultural beliefs link labelled people to undesirable characteristics, (c) labelled people are placed in categories (‘them’ and not ‘us’), and (d) labelled people experience status loss and discrimination [1]. Furthermore, stigmatization is contingent on access to social, economic, and political power that allows the four above components to occur [1]. The general public’s negative beliefs and behaviours are known as public stigma and can contain several beliefs and behaviours as seeing and treating people with mental illness as unintelligent, incapable, dangerous, and blaming or shaming them for their illness [2, 3].

International research shows that public stigma has an adverse impact on life opportunities of people with a mental illness. It is associated with diminished quality of life, social isolation, self-stigma, symptom exacerbation and relapse [4–7]. Furthermore, (anticipated) negative beliefs, exclusion or discrimination may act as a barrier in treatment seeking and for optimal health care for people with mental illnesses [7, 8]. Almost 80% of the people with depression report discrimination on one or more life domains [9], around two-thirds of the people with schizophrenia feel forced to (selectively) hide their diagnosis [10], and a similar proportion anticipates negative discrimination in applying for work, training or education [10].

In order to target and design interventions aimed at the reduction of stigma, it is important to know which living conditions and which characteristics of people with mental illness play a role in stigmatisation [11, 12]. The general picture is that public beliefs and opinions vary over different mental illnesses, with a gradient in rejection depending on the type of the mental illness, where the percentage of respondents endorsing stigmatizing responses generally increases from depression to schizophrenia to alcohol dependence, and finally, to drug dependence [13, 14]. For instance, in a household survey in 2006 in the United States, 74% of the public expressed an unwillingness to work with the individual described in a vignette when it concerned someone with alcohol dependence, against 62% and 47% when the individual in the vignette described had schizophrenia or depression, respectively [15]. Similar patterns are found in more recent studies in different countries, where depicted individuals with schizophrenia elicited more stigmatizing attitudes than individuals with depression [14, 16, 17], and individuals with alcoholism more than individuals with schizophrenia [13, 18]. In addition, familiarity or contact with someone with a mental illness is associated with more positive responses toward people with a mental illness [19–21]. The latter is known to mitigate stigmatizing attitudes due to more knowledge and experience [20, 22]. Having more knowledge on mental health and mental illness is associated with less stigmatizing attitudes towards people with a mental illness [23–25].

Less is known about which characteristics (other than psychiatric diagnosis) or living conditions of people with mental illnesses might affect the likelihood of public stigma. Perkins et al. [26] showed that people with mental illnesses who are employed, elicit less exclusive attitudes than unemployed people. This is both important and a paradox, since stigma is also a serious problem for obtaining and keeping a job in the case of a mental illness [27, 28]. This suggests that effective interventions targeting employment of people with a mental illness, like Individual Placement and Support [29, 30], can have a destigmatizing side-effect, thus further promoting recovery. Therefore it would be useful to see if there are other characteristics of people with a mental illness that might increase or decrease the likelihood of public stigma and if these characteristics interact with the diagnosis of the potentially stigmatized individual.

Next to employment, we focus in this study on the themes youth and housing. Youth is of high importance since mental health problems and mental health stigma can emerge at a young age, and therefore consequences can be drastic [31]. Whether the youth deals with other or more stigma than adults is not known: research on differences in nature or level of stigmatizing attitudes towards either younger or older people is scarce and results are mixed. Speerforck [32] found that the reactions of feeling pity and sympathy were endorsed by significantly more respondents after reading a vignette describing a child with Attention Deficit and Hyperactive Disorder (ADHD), compared to a vignette describing an adult with ADHD. However, for other emotional reactions, like annoyance or anger, no differences in reactions between the vignettes were found. Another study, investigating public stigma towards people with a depression using two different vignettes, found more stigmatizing attitudes towards the depicted younger individual of 25 years old, against the individual of 71 years old [33].

A focus on stigma regarding housing and communities is important, since inclusion of people with a mental illness in their communities contributes to social support, participation and recovery, and often stigma and exclusion emerges in the communities where people with a mental illness live [34–36]. People with mental illness often live in substandard accommodations that are crowded, noisy and located in undesirable neighborhoods [37, 38]. On the one hand, appropriate housing facilities improve the sense of belonging to the neighborhood, and on the other hand: in poor quality neighborhoods, more fear and stigma towards people with mental illness is present [39]. Given the fact that adequate housing, neighborhood order and social cohesion are positively associated with mental health, we are interested in the influence of neighborhood nuisance on stigma [40, 41]. Priority of these themes are acknowledged by the National knowledge consortium on destigmatization in the Netherlands that was established in the spring of 2018 [42].

For this study, we translated the themes employment, youth and housing in characteristics of an individual with a mental illness in (a) being gainfully employed or not, (b) being younger or middle-aged and (c) being the source of domestic noise disturbance or not. Knowing more on social distance associated with such characteristics can serve as an input for developing or stimulating programs aiming at employment, youth and good quality housing, to further empower people with a mental illness. We chose to assess social distance as a measure for stigma because it is seen as one of the core components of stigma and a commonly used for the assessment of the concept [1, 43].

Our hypotheses are that:

1. An individual with a mental illness who is actively engaged in gainful employment will elicit less social distance than an individual with a mental illness that is unemployed;
2. An individual with a mental illness whose neighbors experience no domestic noise disturbance will elicit less social distance than an individual with a mental illness whose neighbors do experience domestic noise disturbance;
3. A young individual with a mental illness will elicit less social distance than a middle-aged individual with a mental illness;
4. An individual with a depressive disorder will elicit less social distance than an individual with a psychotic disorder.
5. An interaction effect for type of disorder on the one hand and unemployment, domestic noise disturbance and older age on the other hand is expected, with the latter characteristics having a stronger negative effect on social distance for a psychotic disorder.

## Methods

### Design, participants and procedure

The study employed a cross-sectional, population-based design. Participants were Dutch citizens recruited from the CentERpanel, a panel set up in 1993 and maintained by CentERdata, which is a Dutch research institute specialized in data collection [44]. The panel is designed to offer an accurate reflection of the Dutch-speaking population. In general, the panel is representative along various dimensions, although small exceptions exist with respect to education (overrepresentation of the upper echelons and underrepresentation of the middle level), household composition (underrepresentation of single households), urbanization (underrepresentation of people living in a highly urbanized setting) and non-western foreigners (including strong underrepresentation on account of language problems and of strong concentration in urban areas) [44].

In January 2018, the 3209 active panel members received a questionnaire. One week after the initial questionnaire invitation, a reminder to complete the questionnaire was sent. One week later, data collection was closed. Respondents completed the questionnaire online, via a secured internet connection on their home computers. Eventually, 2388 panel members started and 12 of them did not complete the procedure, leaving 2376 (74%) questionnaires eligible for analyses. Mean age of the respondents was 54.6 (SD = 16.6), with a minimum of 16 and a maximum of 94 years old; 52% of the respondents were males.

### Measurements

The online questionnaire contained one (from sixteen) randomly assigned vignette describing a fictional male (called by the name of Jeroen, a very common name in the Netherlands) diagnosed with a mental illness and living in the community of a small-sized city. As a city we chose Nieuwegein, a typical Dutch city in the center of the country (similar to Muncie, known from the Middletown Study [45]). To create the descriptions we adapted and extended the vignettes depicting a male with schizophrenia, as used by Perkins et al. [26]. The sixteen vignettes all contained one out of two levels of four variables: (1) diagnosis (a depressive disorder or a psychotic disorder), (2) age (19 years old or 40 years old), (3) causing domestic noise disturbance (present or absent), (4) employment (being employed or not). The vignettes were around 175 words in length (see “Box 1” for an example). As we were mainly focused on the effect of the three variables in the presence of a mental illness, and in the interaction between these, a control vignette for diagnosis (depicting an individual without a mental illness) was omitted.

After reading the vignette, respondents indicated on the Social Distance Scale (SDS) [21, 46] how willing they were for Jeroen to (1) move next door to them, (2) spend an evening socializing with them, (3) make friends with them, (4) start working closely as a colleague with them, and (5) marry into their family. Social distance was rated on a five point scale, with 1 representing ‘definitely not’ and 5 representing the answer ‘definitely’. A middle category was offered as well, with a score of 3 representing the answer ‘maybe’. A total score for social distance was calculated by adding the –reverse coded- answers on the 5-point scale for the five distance levels, resulting in scores varying between 5 (no or very little social distance) and 25 (much social distance). From earlier research, the SDS is known to have good internal consistency [47].

To evaluate the effectiveness of the manipulation in the vignettes, respondents also evaluated Jeroen’s propensity for violence and contribution to his community on a five point scale, with 1 representing ‘very unlikely’ and 5 representing the answer ‘very likely’. It was expected that Jeroen causing domestic noise disturbance would be evaluated at being more prone to violence (compared to Jeroen not causing domestic noise disturbance), and Jeroen being unemployed would be seen as less likely to contribute to his community (compared to Jeroen being employed).

In addition, respondents’ gender, level of contact with people with a mental illness, level of mental health literacy was assessed, and included as covariates in the analyses. Gender was assessed as a standard variable in the CentERpanel. Level of contact was assessed with the Level of Contact Report (LCR), containing seven levels, ranging from having ‘no contact’ with an individual with a mental illness to ‘I do (or did) have a mental illness myself’ [19, 48]. Mental health knowledge was assessed with the MAKS (Mental Health Knowledge Schedule), a 12 item questionnaire containing six stigma-related mental health knowledge areas: help seeking, recognition, support, employment, treatment, and recovery, and six items that inquire about knowledge of mental illness conditions. Response categories vary between (1) ‘strongly disagree’ to (5) ‘strongly agree’, total scores range between 12 and 60, with a higher score indicating more mental health knowledge. Although earlier research showed that the overall internal consistency of the MAKS was moderate [49], to our knowledge, no other short instrument covering mental health knowledge was available.

Box 1 Example of a vignette of Jeroen (middle aged, the source of domestic noise disturbance, depressive disorder, unemployed). Italics indicate text that differ for the levels of the four variables.

Jeroen is *a 40 year old man* and lives in an apartment in Nieuwegein. After finishing school he started working as a logistic employee. After a few years he started *to feel down, often for longer times. He had no appetite and lost quite some weight. His ability to concentrate on daily activities disappeared, as well as the energy to undertake any outings with his girlfriend. When he lost his job, he felt even more useless and he started suffering from a feeling of guilt and insomnia.* Jeroen is often awake at night. *Sometimes the neighbors complain about noise disturbance.* A year ago his brother convinced him to seek help at a local mental health organization, where he was diagnosed with a *depressive disorder*. He has been taking medication ever since, next to group therapy. *At this moment, Jeroen is unemployed and often visits the library to read some magazines.*

### Analyses

Descriptive statistics were analyzed with frequencies and means. For the evaluation the effectiveness of the manipulation in the vignettes, *t* tests were performed. Gender, level of contact (LCR) and mental health knowledge (MAKS) of the respondents were included as covariates, and their univariate associations with the outcome (the aggregated SDS-score) were analyzed with an one-way ANOVA and bivariate pearson correlations. To test the five hypotheses, a four-way ANOVA was performed, with age, employment, domestic noise disturbance and diagnosis as main effects. In addition, gender, the LCR and MAKS scores added as covariates in the four-way ANOVA.

## Results

Results of the manipulation check showed that respondents who read the vignettes in which Jeroen caused domestic noise disturbance, evaluated him as being more prone to violence towards others (*M* = 2.58; SD = 0.88), compared to respondents that read the vignette in which Jeroen did not cause any disturbance (*M* = 2.45, SD = 0.90; *t* (2374) = 3.50, *p* < 0.01; *d* = 0.15). In addition, respondents that read the vignettes in which Jeroen was employed, saw him as being more likely to contribute to his community (*M* = 3.51; SD = 0.88), compared to respondents that read the vignette in which Jeroen was not employed (*M* = 2.70, SD = 0.90; *t* (2374) = 22.40, *p* < 0.01; *d* = 0.91).

Bivariate correlations between, and means, percentages (for different categories) and effect sizes of the covariates and study variables are shown in Table 1. Distribution of the covariates over the vignettes showed no differences for the MAKS-score and gender. For the LCR score differences were found [*F*(15, 2360) = 2.50, *p* < 0.01]. As can be seen in Table 1, respondents that read the vignettes with Jeroen having a psychosis is associated with noise disturbance and is 40 years of age have lower mean LCR scores than respondents that read vignettes with the other level of these variables.

**Table 1:** Correlations, means, effect sizes, percentages (for different categories). and four-way ANOVA statistics for covariates and study variables

	Gender		MAKS-score	LCR-score	SDS-score	ANOVA		
	Female (%)	Male (%)				F-ratio	d*f*	*η*^2^
Respondent characteristics ↓								
Gender								
Female			45.2 (4.96)**^1^	3.5 (2.49)**^2^	15.0 (3.88)**^3^	4.79*	1. 2357	0.002
Male			43.9 (4.95)	3.1 (2.50)	15.6 (3.88)			
MAKS-score			–	0.360**	− 0.216**	50.8*	1. 2357	0.021
LCR-score			0.360**	–	− 0.232**	61.9*	1. 2357	0.026
Characteristic of vignette’s individual ↓								
Age								
19 years	51.8	50.4	44.6 (4.98)	3.34 (2.47)*^4^	15.2 (3.90)	0.023	1. 2357	0.000
40 years	48.2	49.6	44.4 (5.02)	3.14 (2.53)	15.4 (3.88)			
Noise disturbance								
Yes	50.6	48.2	44.4 (5.03)	3.08 (2.48)**^5^	15.6 (3.93)**^6^	0.483*	1. 2357	0.003
No	49.4	51.8	44.7 (4.97)	3.41 (2.51)	15.0 (3.83)			
Employed								
Yes	48.6	51.7	44.5 (5.09)	3.20 (2.51)	14.8 (3.99)**^7^	55.8*	1. 2357	0.023
No	51.4	48.3	44.6 (4.91)	3.29 (2.49)	15.8 (3.71)			
Diagnosis								
Psychosis	51.6	50.8	44.4 (4.98)	3.10 (2.51)**^8^	15.9 (3.76)**^9^	51.8	1. 2357	0.022
Depression	48.4	49.2	44.6 (5.01)	3.40 (2.48)	14.7 (3.93)			

Superscripts indicate Cohen’s *d*: 1 = 0.26; 2 = 0.16; 3 = 0.16; 4 = 0.08; 5 = 0.13; 6 = 0.16; 7 = 0.26; 8 = 0.12; 9 = 0.31

*p < 0.05

**p < 0.01

**Fig. 1:**
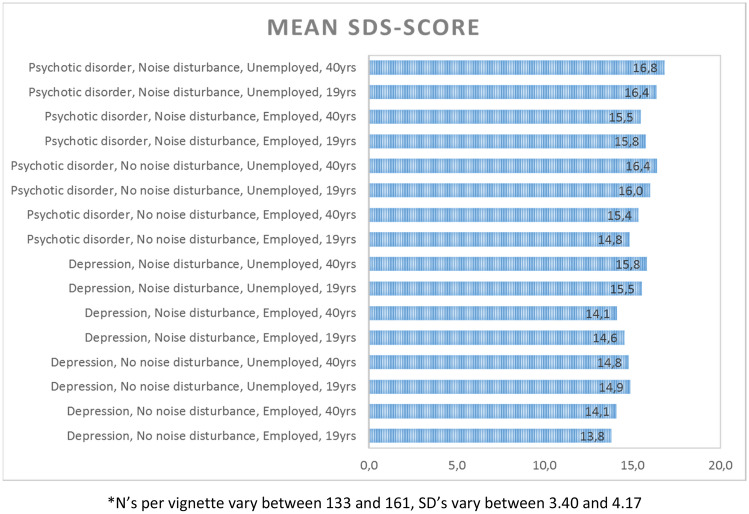
Mean SDS-scores vignettes. *N’s per vignette vary between 133 and 161, SD’s vary between 3.40 and 4.17

Mean SDS-score for all vignettes are shown in Fig. 1. The aggregated mean SDS-score -covering all levels of the four variables- was 15.3 (SD = 3.89). Regarding covariates, mean SDS-scores were lower for females, for respondents with a closer level of contact according to the LCR, and for respondents with a higher MAKS score (see Table 1).

The four-way ANOVA without covariates, yielded main effects for employment (*F*(1, 2360) = 47.65, *p* < 0.01, *η*^2^ = 0.020), domestic noise disturbance [*F*(1, 2360) = 12.0, *p* < 0.01, *η*^2^ = 0.005] and diagnosis [*F*(1, 2360) = 56.8, *p* < 0.01, *η*^2^ = 0.023] on the SDS-score. No main effect for age and no-interaction effects were found. Explained variance of the model was 5.1% (*R*^*2*^ = 0.051). Table 1 shows results for the four-way ANOVA with covariates, with main effects remaining similar and effect sizes to be classified as small [50]. Explained variance of this model was 12% (*R*^*2*^ = 0.121).

Jeroen being employed elicited less social distance, as well as Jeroen not being associated with domestic noise disturbance and having a depressive disorder. The vignette in which Jeroen has a depressive disorder, is employed, is not associated with domestic noise disturbance and is 19 years old elicited the lowest SDS-score (*M* = 13.8, SD = 4.02), whereas the vignette in which Jeroen has a psychotic disorder, is unemployed, is associated with domestic noise disturbance and is 40 years of age, elicited the highest SDS-score (*M* = 16.8, SD = 3.66). Translated to actual answers of respondents, this means that 33.3% percent of the respondents answered ‘no’ on the question ‘Would you like Jeroen to spend an evening socializing with you?’, for Jeroen, 40 years of age with a psychotic disorder, who is unemployed and is associated with domestic noise disturbance. Whereas 17.5% of the respondents answered similar for Jeroen with a depressive disorder, who is employed, is not associated with domestic noise disturbance and is 19 years old. For the willingness of Jeroen marrying into the family, the differences were sharper: 68.0% ‘no’ vs. 32.4% ‘no’ for the vignettes described above, respectively.

## Discussion

Unemployment and an association with domestic noise disturbance of a fictional individual with a mental illness were independently associated with increased stigma, as measured with the SDS. Also, in line with other research, stigma is stronger for a psychotic disorder compared to a depressive disorder [13]. Age of the fictional individual was not associated with the level of stigma. Not in line with our hypothesis, unemployment and domestic noise disturbance did not interact with the type of mental illness, indicating that the stigmatizing effects of these characteristics are of similar strength for people with a depressive disorder and a psychotic disorder. Furthermore, level of contact and mental health knowledge are negatively associated with stigma.

Social distance was about the same for Jeroen with a psychotic disorder, with domestic noise disturbance and employment, as for Jeroen with a depressive disorder (and domestic noise disturbance) without employment. Similar patterns were found in the study of Perkins [26], suggesting that one single characteristic can mitigate a stereotype that people may hold of people with a mental illness.

Strength of the study is the high response on the questionnaire, resulting in a relatively accurate reflection of the Dutch-speaking population in the Netherlands and sufficient power to study subgroups or correct for other associations (as we did in adding gender, level of contact, mental health knowledge as covariates). Simultaneously, resulting in a disadvantage, this sample size also yields that even very small differences or effects reach significance very easily, as is the case in this study. Additional limitations of the study are the absence of a ‘control’ vignette: the situation in which the fictional persona has no mental illness. This prevents taking conclusions about an absolute effect of characteristics on stigma. Furthermore, we used the MAKS as an unidimensional scale for mental health knowledge. As indicated, the reliability of this scale was modest, and therefore results based on this use of the MAKS should be interpreted with caution. More research and attention to the use of the MAKS to assess mental health knowledge is warranted. Lastly, the reader should be aware that the variance explained by the model was a modest 12%, and effect sizes were (very) small. This means that offering additional, potentially destigmatizing information about people with mental illnesses only slightly alters someone’s perception. For the effect of noise disturbance the practical relevance is questionable since its effect neared 0%.

Our results suggest that supporting clients in getting and keeping gainful employment can have a positive effect on the process of destigmatisation and social inclusion of people with a mental illness, by directly reducing the negative perceptions held by the general public. We also showed that this is independent of the diagnosis of the potentially stigmatized individual: it can be equally effective for people with diagnoses that differ in the strength of stigmatization. We also showed that this effect is independent of gender, level of contact and knowledge about the mental health of the general public.

These findings underline the importance, added value and paradox-solving of methods like Individual Placement and Support (IPS), which is effective in helping people with severe mental illnesses to find competitive employment, and is implemented in many countries [30, 51, 52].

As mentioned, the practical relevance of intervening in noise disturbance seems questionable given the small effect size in this study. However, the SDS contains just one question touching the topic of ‘living next door’. The other questions in the scale imply a closer contact, where the element of ‘noise disturbance’ is might be less relevant. This might have resulted in a relatively small opportunity to get the effect of noise disturbance expressed via a total score on the SDS. Further research should reveal if this remains the case. Additional implications for further research involve investigation of the generalizability of these findings toward other diagnoses, like drug or alcohol dependence. This is important, since in general—and in this study as well—the gradient of desired social distance follows ‘the more intimate the setting, the more likely the desired social distance’, but the gradient is not neat: for drug and alcohol dependence, ‘living next door’ produced a greater stigmatizing response than ‘friendship’ in social distance scale terms [13]. Also, investigating generalizability of the current findings towards other samples in other countries would be of interest, although the concordance with results of Perkins’ study [26] suggests the current results being applicable to more settings, next to the finding that regardless of the presence of a mental illness, unemployment elicits more negative attitudes [53, 54]. Next, although in this study included as a covariate: design, scoring and scaling of the MAKS as a measure for mental health should be subject of further research, especially because improving knowledge can be a relatively feasible and successful method to lessen public stigma, as the effect size in this study indicates as well. This would yield more research on the level of mental health knowledge, and about correlates of mental health knowledge and stigma, that appears to be negatively associated (the more knowledge, the less stigma) [24]. Lastly, the absence of a ‘control’ vignette (the situation in which the fictional individual has no mental illness) in this study calls for a study that includes one, allowing conclusions about an absolute effect of characteristics on social distance.

## Data Availability

Not applicable
